# Enhanced frequency of neutrophils and inflammatory monocytes and diminished numbers of T and B cells in active pulmonary tuberculosis

**DOI:** 10.1186/1471-2334-12-S1-P23

**Published:** 2012-05-04

**Authors:** N Pavan Kumar, Luke Elizabeth Hanna, MS Jawahar, VV Banu Rekha, R Sridhar, Thomas B Nutman, S Subash Babu

**Affiliations:** 1 National Institutes of Health – International Center for Excellence in Research, Chennai, India; 2 National Institute for Research in Tuberculosis, Chennai, India; 3 Laboratory of Parasitic Diseases, National Institutes of Allergy and Infectious Diseases, National Institutes of Health, Bethesda, Maryland, USA; 4 Government Stanley Medical College and Hospital, Chennai, India

## Background

*Mycobacterium tuberculosis* (M.tb) infects nearly 2 billion people worldwide. Effective immunity against M.tb infection requires co-ordinated responses from both innate and adaptive arms of immunity. To elucidate the immune responses important both in control of infection and in extra-pulmonary dissemination, we examined frequency and/or absolute numbers of T, B and NK cells, dendritic cells and other leucocyte populations in active tuberculosis patients.

## Methods

The frequency as well as absolute numbers of T cells (CD3+, CD4+, CD8+ T cells), B cells and NK cells as well as the frequency of innate immune cells (neutrophils and monocytes), dendritic cell subsets (pDC & mDC ), T cell subsets (naïve, central and effector memory and regulatory T cells) was examined by flow cytometry in AFB smear positive pulmonary TB (Sm+) (n=30) and AFB smear negative pulmonary TB (Sm-) (n=24) and compared with extra- pulmonary TB (EP) (n=38).

## Results

Among the innate immune subsets, we observed significantly higher frequency of neutrophils and inflammatory monocytes in Sm+ pulmonary TB group when compared with Sm- pulmonary and EP TB group. On the other hand, the absolute numbers of CD3+ T cells, CD4+ T cells, CD8+ T cells and B cells were significantly lower in Sm+ when compared with Sm- and EP TB group.

## Conclusion

Pulmonary TB is characterized by enhanced frequencies of neutrophils and inflammatory monocytes and diminished absolute counts of T and B cells, suggesting a crucial role for these cell populations in protection against TB disease development as well as extra-pulmonary dissemination.

